# Supplementary material for: Dynalign II: common secondary structure prediction for RNA homologs with domain insertions
Source: Nucleic Acids Res. 2014 Nov 21;42(22):13939–48. doi: 10.1093/nar/gku1172 (PMC4267632; doi:10.1093/nar/gku1172)
Supplement: SUPPLEMENTARY DATA [file supp_gku1172_nar-02021-z-2014-File012.zip › manual/Text/ProbablePair.html]

RNAstructure Command Line Help: ProbablePair


|  |  |  |
| --- | --- | --- |
|  | RNAstructure Command Line Help ProbablePair | - Contents - Index |
| ProbablePair creates a group of suboptimal structures that contain base pairs above a probability threshold. USAGE: ProbablePair <input file> <ct file> [options] Required parameters: <input file> \| The name of a file containing input data. This input data can be in one of two formats: 1. Partition function save file (holds base pairing probability data for all pairs and can be generated using partition). 2. Sequence file (holds sequence: .seq or .fasta). Note that lowercase nucleotides are forced single-stranded in structure prediction. Note that in order to use a squence file, the "sequence" flag must be specified (see "--sequence" below).; <ct file> \| The name of a CT file to which output will be written. Options which don't require added values: -d, -D, --DNA \| This flag only matters if the input file is a sequence file and has been identified as such (see "--sequence" below). Specify that the sequence is DNA, and DNA parameters are to be used. Default is to use RNA parameters.; -h, -H, --help \| Display the usage details message.; --sequence \| Identify the input file format as a sequence file. Options which require added values: -t, -T, --threshold \| Specify the pairing threshold for pairs to be included. This should be expressed as a number >= 0.5 and <= 1. Default is 0, which signifies structures should be generated at multiple thresholds: 0.99, 0.97, 0.95, 0.90, 0.80, 0.70, 0.60, and 0.50. References: 1. Reuter, J.S. and Mathews, D.H. "RNAstructure: software for RNA secondary structure prediction and analysis." *BMC Bioinformatics*, 11:129. (2010). 2. Mathews, D.H. "Using an RNA secondary structure partition function to determine confidence in base pairs predicted by free energy minimization." *RNA*, 10:1178-1190. (2004). | | |
| Visit The Mathews Lab RNAstructure Page for updates and latest information. | | |
